# Optimal Growth Temperature and Intergenic Distances in Bacteria, Archaea, and Plastids of Rhodophytic Branch

**DOI:** 10.1155/2020/3465380

**Published:** 2020-01-17

**Authors:** Vassily A. Lyubetsky, Oleg A. Zverkov, Lev I. Rubanov, Alexandr V. Seliverstov

**Affiliations:** Institute for Information Transmission Problems of the Russian Academy of Sciences (Kharkevich Institute), Bolshoy Karetny 19, Moscow 127051, Russia

## Abstract

The lengths of intergenic regions between neighboring genes that are convergent, divergent, or unidirectional were calculated for plastids of the rhodophytic branch and complete archaeal and bacterial genomes. Statistically significant linear relationships between any pair of the medians of these three length types have been revealed in each genomic group. Exponential relationships between the optimal growth temperature and each of the three medians have been revealed as well. The leading coefficients of the regression equations relating all pairs of the medians as well as temperature and any of the medians have the same sign and order of magnitude. The results obtained for plastids, archaea, and bacteria are also similar at the qualitative level. For instance, the medians are always low at high temperatures. At low temperatures, the medians tend to statistically significant greater values and scattering. The original model was used to test our hypothesis that the intergenic distances are optimized in particular to decrease the competition of RNA polymerases within the locus that results in transcribing shortened RNAs. Overall, this points to an effect of temperature for both remote and close genomes.

## 1. Introduction

The dependence of intergenic distances between each other and on the species' optimal growth temperature was considered in three large groups: plastids of the rhodophytic branch, archaea, and bacteria. For consistency, we use the term *group* in these three cases, although the group of plastids is not taxonomic. We distinguish three types of neighboring gene arrangements: convergent (⟶⟵), divergent (⟵⟶), and unidirectional (⟶⟶ or ⟵⟵). Median is the middle value of a series of numbers arranged in ascending order. The median proves better than the arithmetic mean since few very long intergenic regions substantially increase the mean but have a lesser effect on the median. A significant correlation between intergenic distances in convergent and divergent neighboring gene pairs was observed in several archaea and bacteria [1]. We continue the studies on the distance between neighboring genes initiated previously, e.g., in [2]. Let the abbreviation OGT means the middle of the optimal growth temperature range. The considered OGTs range from 2 to 52.5°C in plastid species, from 22.5 to 114°C in archaea, and from 7 to 85°C in bacteria (ref. to the Supplementary Spreadsheet) ([Supplementary-material supplementary-material-1]). Usually, the growth temperature range is much wider than the range of optimal temperatures. For example, the growth temperatures of eukaryotic algae range from −7°C for Antarctic algae [3] to 56°C for Cyanidiophyceae algae living in hot springs. Many algal species live at relatively low temperature.

Plastids are semiautonomous organelles originating from cyanobacteria; in the rhodophytic branch, they are represented in red algae (Rhodophyta) as well as in species with plastids of secondary or tertiary origin from Rhodophyta plastids. Such species belong to the superphyla Alveolata and Heterokonta (classes Bacillariophyceae, Bolidophyceae, Chrysophyceae, Dictyochophyceae, Eustigmatophyceae, Phaeophyceae, Xanthophyceae, and Raphidophyceae) as well as to the phyla Cryptophyta and Haptophyta. Plastids of brown, diatom, yellow-green, and other related algae grouped into Stramenopiles also have secondary origin from plastids of red algae [4]. Tertiary plastids acquired from diatoms have been found in alveolates *Durinskia baltica* and *Kryptoperidinium foliaceum* [5]. Descendants of Rhodophyta plastids also include apicoplasts, plastids of parasitic apicomplexans [6, 7]. The plastids encode only several transcription factors [8], while numerous nucleus-encoded transcription factors are imported to plastids from the cytoplasm.

Archaea are prokaryotes distinct from bacteria and are putative ancestors of eukaryotes [9–12]. Archaea are widely distributed [13–15] living in diverse environmental conditions. This makes archaea a convenient model to study the impact of environmental factors on the genome structure. Their optimal growth temperature varies in a wide range, from 20°C to 116°C. Thermophilic archaea include representatives of early diverged phylogenetic groups Crenarchaeota and Euryarchaeota. Although certain species can grow at 4°C, the optimal growth temperature is above 30°C for most archaea. *Methanoculleus marisnigri* (20–25°C) and *Methanococcoides burtonii* (23.4°C) are the exceptions among the considered species.

Taking into account the cyanobacterial origin of rhodophytic plastids, the considered bacteria include no cyanobacteria except *Crinalium epipsammum* and *Dactylococcopsis salina*. Many bacteria live under the same conditions as archaea [16]. The bottom growth temperature is lower in bacteria than in archaea. Bacteria can grow at temperatures from −2°C (*Cellulophaga algicola*) to 95°C (*Aquifex* genus). However, their OGTs are positive and do not exceed 85°C (*Aquifex aeolicus*). The OGTs can vary even between closely related species of the same genus. Such pairs of species include *Bacillus methanolicus* and *B. subtilis*; *Cellulophaga algicola* and *C. lytica*; *Lactobacillus amylolyticus* and *L. lindneri*; *Desulfotomaculum acetoxidans* and *D. nigrificans*; *Acidithiobacillus caldus* and *A. ferrooxidans*; *Shewanella oneidensis* and *Sh*. *violacea*; and *Spirochaeta africana* and *Sp. thermophile* (ref. to the Supplementary Spreadsheet).

## 2. Results

A genome is described here by three medians for each type of gene arrangement: convergent, divergent, and unidirectional. These medians are referred to as *con*, *div*, and *uni*, respectively (or by their first letters *c*, *d*, and *u*). These three medians as well as the OGT (designated as *T*) are given for each species and strain in the Supplementary Spreadsheet.

### 2.1. Correlations between Medians

**Figure 1:**
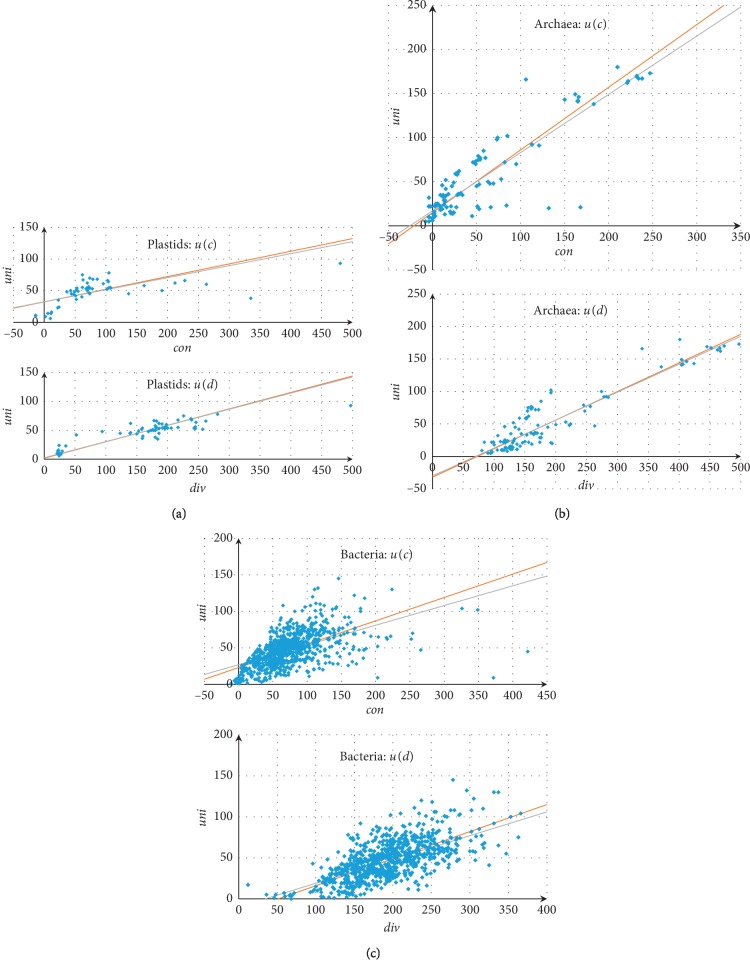
Linear regression plots for pairs of medians in plastids (a), Archaea (b), and Bacteria (c). Simple regressions are shown in gray and Deming regressions in red. Negative values are due to overlapped genes.

The correlation coefficients for *con*, *div*, and *uni* in all three groups indicate a significant correlation between the medians (Supplementary Materials [Supplementary-material supplementary-material-1]). The best correlations themselves are represented by simple and Deming linear regressions independently for each of the three groups in Figure 1. Specifically, for plastids *u* = 0.19*c* + 32.5 (*u* = 0.20*c* + 32.3), *u* = 0.28*d* + 2.5 (*u* = 0.29*d* + 1.7); for archaea: *u* = 0.66*c* + 17 (*u* = 0.71*c* + 15), *u* = 0.43*d*–30 (*u* = 0.44*d*–32); for bacteria: *u* = 0.27*c* + 27 (*u* = 0.32*c* + 23), *u* = 0.29*d*–10 (*u* = 0.33*d*–17). Shown in parentheses are Deming regressions; see more details in Supplementary Materials [Supplementary-material supplementary-material-1]. For convergent genes, the medians are typically greater than those for divergent and unidirectional ones.

### 2.2. Clustering by Temperature

Genome assignment to one of the thermal ranges is indicated by a number in the Supplementary Spreadsheet (column *G*). Thus formed subsets (parts) of the species are the same for each of the three medians *con*, *div,* and *uni*, which is by no means evident from general considerations.

The analyzed plastids (59 genomes in total) excluding that of *Karlodinium veneficum* can be clustered into four parts by OGT that fall into intervals: 0–15°C (12), 15–20°C (13), 20–30°C (22), and 30–55°C (11); the number of genomes in each part is given in parentheses. The Fisher index values *F*_calc_ (see Supplementary Materials [Supplementary-material supplementary-material-1]) for significant difference between the mean values of these parts for the three medians are 4.6, 22, and 30, respectively; the latter two are substantially greater than the tabular value *F*_*α*_ (*k − *1, *n − k*) = *F*_0.05_(3, 54) = 2.8. The plastid in *K. veneficum* has untypically high *con* = 1090; if included, the Fisher index becomes equal to 2.5, which is below the tabular value.

The considered archaea (123 genomes) can be clustered into three parts by OGT, which is natural since little data are available for archaea living at temperatures below 30°C (in contrast to plastids). These intervals are 20–40°C (47), 40–65°C (15), and 65–115°C (61). The corresponding Fisher indices *F*_calc_ equal 46, 40, and 40; all of them are much higher than the tabular value *F*_0.05_(2, 120) = 3.1.

The considered bacteria (810 genomes) can also be clustered into four parts by OGT with the following intervals: 5–30°C (305), 30–40°C (406), 40–65°C (45), and 65–85°C (54). The corresponding Fisher indices *F*_calc_ equal 18, 12, and 31, which are much higher than the tabular value *F*_0.05_(3, 806) = 2.6. The proposed clustering of species by temperature *T* is in a good agreement with the traditional classification into hyperthermophiles thermophiles, mesophiles, and psychrophiles. They are approximately as follows: above 60°C, above 45°C, in 20–45°C, and below 10°C, respectively. A comparison of the partition parts based on different indices is provided in Supplementary Materials [Supplementary-material supplementary-material-1]. The temperature clustering indicates a temperature-median relationship, which is considered below.

### 2.3. Correlations between Medians and OGT

**Figure 2:**
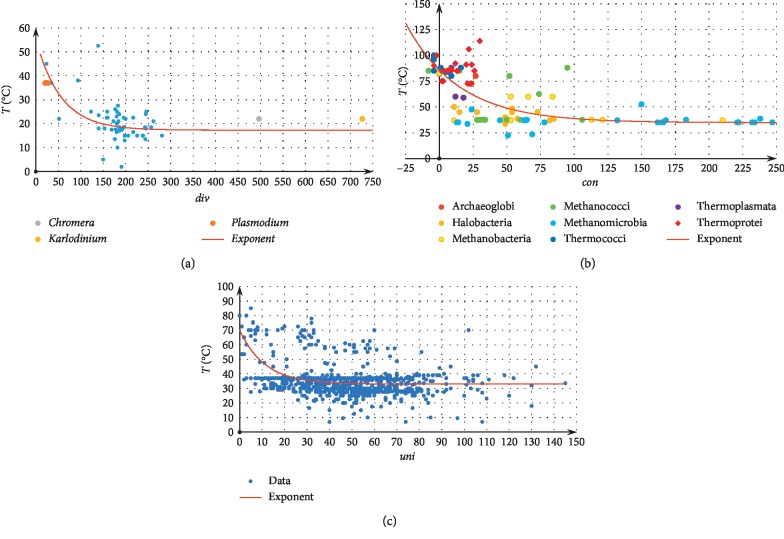
Scatter plots for temperature versus medians and the corresponding nonlinear regressions for plastids (a), archaea (b), and bacteria (c).

In all groups, the best regressions between each of the medians and OGT (designated as *T*) are exponential. For short, in Figure 2, we draw one regression for each group: *T*(*div*) for plastids, *T*(*con*) for archaea (negative values are due to overlapped genes), and *T*(*uni*) for bacteria. More detailed data on the regressions for different classes of functions are provided in Supplementary Materials [Supplementary-material supplementary-material-1]. Leading coefficients in the exponent indices of the computed regressions are similar for different types of gene arrangement and groups. Plastids: *T*=20.3 exp{−0.022*c*}+16.6, *T*=38 exp{−0.018*d*}+17.3, *T*=21 exp{−0.035*u*}+18.1; Archaea: *T*=50.9 exp{−0.0256*c*}+34.8, *T*=262 exp{−0.0155*d*}+34.8, *T*=58.2exp{−0.0178*u*}+31.7; Bacteria: *T*=22 exp{−0.075*c*}+34, *T*=41 exp{−0.015*d*}+32, *T*=37 exp{−0.089*u*}+33.

### 2.4. Modeling for Convergent Genes

**Figure 3:**
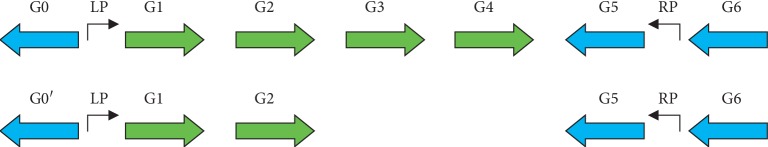
A locus in *S. africana* (upper) and *S. thermophila* (lower): a relationship between intergenic distance and optimal environmental temperature. The syntenic region includes six genes (G1 to G6) coding for large and small subunits of acetolactate synthase (G1 and G2), dihydroxy-acid dehydratase (G3), lysine 2,3-aminomutase (LAM) encoded by the gene kamA, a member of the radical SAM superfamily (G4), tRNA-Val (G5), and a response regulator (G6). Nonorthologous genes G0 and G0*′* code for phytoene desaturase and DUF401 domain-containing protein, respectively. *LP* and *RP* are potential promoters.

We assume that at high temperature the distance between genes is generally smaller than that at low temperature. As an example, let us consider a pair of species *Spirochaeta africana* and *S. thermophila* as well as their loci encoding valine tRNA and the enzymes for biosynthesis of branched-chain amino acids (Figure 3). For *S. africana*, the growth temperature range is 15–47°C and the optimal temperature range is 30–37°C. The corresponding values for *S. thermophila* are 40–73°C and 66–68°C, respectively. These loci contain a syntenic region with the genes encoding two subunits of acetolactate synthase followed by the convergently arranged gene encoding valine tRNA with a GAC anticodon. The exact location of promoters is not available for these loci. Apparently, one of the promoters (designated as LP) lies between the divergent genes G0 (or G0′) and G1, and the other promoter (designated as RP) lies between G5 and G6 since G5 should be actively transcribed. The specific binding to promoters upstream of RP is taken into account for the intensity of binding to RP.

*S. africana* has two genes that are missing in *S. thermophila* between the genes encoding the subunits and the tRNA gene. This can be interpreted as a significant decrease in the distance between the genes encoding the subunits and the tRNA gene in a species living under higher temperatures. Notice that we assumed that the genome structure is fixed here, although it can sometimes change with environmental conditions including temperature and concentration of substances. This can be exemplified by the inversion of the *fimS* region that is located upstream of the *fimA* gene in *E. coli* [17]. Generally speaking, the number of copies of a tRNA species is higher than the number of copies of an mRNA species: an mRNA can be used to synthesize many protein copies, while tRNAs are directly involved in translation and participate in the activity of many ribosomes. Under permissible temperature, the genes encoding the acetolactate synthase subunits and the tRNA are actively transcribed; the subunits are synthesized in roughly equimolar amounts.

Now let us consider the ratio *X* of transcription levels of the subunits (G2 to G1) and the ratio *Y* of transcription levels of tRNA and the first subunit (G5 to G1). The following *condition* is considered: *X* and *Y* are greater than the predefined thresholds varied in modeling. Our model of interaction between RNA polymerases [18, 19] evaluates transcription levels for all genes in a locus of interest. The level is defined as the number of gene transcription events within a fixed time period (3 h in this case). This model was used to determine the rates of RNA polymerase binding to the four promoters satisfying the above condition for different threshold levels; the resulting four numbers will be referred to as a *solution*.

The model requires that the elongation rate of RNA polymerase is specified, which is not known precisely from experiments. Ryals et al. [20] specified five values for *E. coli* (temperature, centigrade-elongation rate, nt/s): (20.5, 32), (25, 45), (30, 59), (37, 86), and (42, 118). The value for 37°C is in good agreement with the estimate of 79–91 nt/s in [21]. At the same time, Abbondanzieri et al. [22] specified other temperature-specific rates. Instead, we used the above five “temperature-elongation rate” points approximated by an exponential curve *v* = 9.8881·exp(0.059*T*), which allows us to apply the model. Other parameters have default values. The modeling was made on a grid with the intensity of binding attempts to each promoter from 0.001 to 1.9 s^−1^, the nodes of which are close to geometric progression with a ratio of 1.08.

The boundaries of growth and optimal temperatures as well as lethal or sublethal (suppressing growth) temperatures (marked by an asterisk) were considered for both bacterial species: *S. africana*, 15, 30, 37, 47, and 67^*∗*^; *S. thermophila*, 20^*∗*^, 40, 67, and 73. Similar results are observed for close temperatures.

In the case of *S. thermophila*, there is exactly one solution for each admissible temperature where similar transcription levels are observed for two subunits of acetolactate synthase (*X* > 0.78), while the ratio between the levels of tRNA and the first subunit is high (*Y* > 3). For instance, the levels of these RNAs equal 63, 53, and 198 at 40°C; 324, 270, and 1000 at 67°С; and 542, 422, and 1998 at 73°С. At these temperatures, the intensity of promoter binding is 1.9 times higher for LP than for RP, while for RP it equals 0.01, 0.05, and 0.1 s^−1^, respectively. There is not any solution at 20°С for the same thresholds for *X* and *Y*. Thus, the model yields sensible solutions for *S. thermophila*.

A rather different situation is observed for *S. africana*. For instance, on condition that *X* > 0.7 and *Y* > 3, we have 15 solutions in total for all admissible temperatures as well as 5 solutions for inadmissible temperatures. The solutions for the same thresholds for *X* and *Y* in *S. thermophila* are different: 8 solutions in total for all admissible temperatures and not any solution for inadmissible ones. At the same time, the solutions for inadmissible temperatures in *S. africana* can stem from specific properties of this locus and, in any case, do not conflict with the assumption mentioned at the beginning of this subsection.

Also important are the mechanisms that coordinate intergenic distances and optimal temperature of the species on the proportion of 1D to 3D diffusion rates of RNA polymerases. Proteins that act at specific DNA sequences initially bind random DNA and then translocate to the target site. Proteins move along DNA by multiple cycles of dissociation and reassociation with the same DNA. Each landing at a new site is then followed by a series of one-dimensional diffusion steps covering 50–100 bp around a site [23–25]. According to Gowers et al. [26], a protein at 37°C can slide along DNA between sites only when the sites are less than 50 bp apart. Transfers over longer distances always include at least one dissociation step. Hence, at higher temperatures, one-dimensional diffusion occurs only over short distances, while three-dimensional diffusion from dissociations to reassociation is the main mode of protein translocation.

This suggests that the proportion between the contributions of 1D diffusion and 3D diffusion to the recognition of specific DNA binding sites by both RNA polymerases and transcription termination factors can be important at least in the case of divergent and unidirectional genes. Thus, 1D diffusion matters at low temperature and long intergenic regions serve as an “antenna” assembling the proper RNA polymerase and transcription factors. At high temperature, 1D diffusion is not as significant and this antenna is not needed; moreover, a high elongation rate allows RNA polymerase to transcribe long operons using a single promoter without additional ones.

## 3. Discussion

A linear relationship between the medians is beyond question; the corresponding linear regressions are unconditionally confirmed by the statistical test for plastids, archaea, and bacteria. Analysis of the relationship between the medians and the temperature *T* confirms both exponential dependence and hyperbolic dependence; the difference between them is minor in the range of definition. The residuals conform to the normality property (a normal distribution) for plastids, partially for archaea, but not for bacteria (Supplementary Materials [Supplementary-material supplementary-material-1]). Suboptimal regressions for plastids and archaea feature the normality, which we consider as advantageous over optimal regressions. No normality is observed in bacteria for both optimal and suboptimal regressions (Supplementary Materials [Supplementary-material supplementary-material-1]).

Alterations of environmental temperature (resulting from changed habitat or climate) underlie the predominant survival of organisms with the median intergenic distances fitting the temperature. Our hypothesis is the following. Evolutionary selection provides for the adaptation of the medians to environmental temperature so that high temperatures and small medians as well as low temperatures and large medians favor efficient survival in at least three considered groups. Indeed, the relationship between the medians and temperature is quite similar for distant groups: plastids, archaea, and bacteria; which points to a macroevolutionary effect of temperature. Adaptation to temperature also proceeds at the microevolutionary level: a locus was exemplified in close species *S. africana* and *S. thermophila* where the intergenic distance changed together with the environmental temperature. At the same time, each of the groups includes specific exceptions, which are considered below. For instance, such deviation is observed for smaller medians in archaea, while in bacteria the deviation is scattered more or less evenly throughout the data range. Here, one can see the influence of other stable environmental factors. These exceptions reflect both the environmental effect (temperature) and phylogenetic conservatism. A similar hypothesis for bacteria not accounting for relative orientation of genes has been proposed elsewhere [27].

Although at high temperatures only small median values are observed, the species living at relatively low temperatures feature the more dispersed medians.

### 3.1. Plastids

A significant deviation from the general pattern is observed in the plastids of phototrophic alveolates: *Chromera velia* and *Kryptoperidinium foliaceum* (Figure 1). This can be due to a relatively recent acquisition of plastids.

The plastids of algae of the family Bangiaceae with a complex temperature-dependent life cycle have the distances (with an account of all three types, see the sheet “Bangiaceae” in the Supplementary Spreadsheet) similar to those in other algae living at low temperature (Figure 1 and the corresponding regressions). This allows us to propose that the gene expression in their plastids is critical at low temperatures during the active growth of the blades. It is hard to determine the OGT for Bangiaceae and Florideophyceae due to their specific ontogeny. The optimal temperature for conchosporangia formation is 25°C in *Porphyra yezoensis* HB (*Pyropia yezoensis*) [28]. In *P. columbina*, conchosporangia are formed at 10–15°C depending on the light period, while the growth of juvenile fronds peaks at 15°C [29]. The freshwater alga *Batrachospermum turfosum* (Florideophyceae) is capable of net photosynthesis at temperatures between 5°C and 35°C [30].

Large distances between genes in the plastids of *Chromera velia* can be attributed to numerous structural rearrangements of their chromosomes [31].

For all types, the small medians in the apicoplasts of *Leucocytozoon caulleryi* and *Plasmodium* spp. confirm that the apicoplasts are crucial for the infection of warm-blooded host cells: the apicoplast is reactivated by the infection as its distances are adjusted to the host temperature. This confirms the previous results on the role of apicoplasts [32, 33]. On the contrary, *Toxoplasma gondii* and other considered coccidians do not occur in hosts living at low temperatures.

### 3.2. Archaea

The medians take on only small values at high temperatures (Figure 1). Species living at relatively low temperatures can have substantially different medians.

The results obtained for plastids of the rhodophytic branch are qualitatively similar to those for archaea. However, there are significant distinctions, in particular, the minimum standard deviation, coefficients of the corresponding regressions, and the proper medians for three types of gene arrangement markedly differ.

Only minor differences in temperature and the medians are observed within the classes Archaeoglobi, Halobacteria, and Thermococci (Figure 1). Representatives of Thermoprotei live under significantly different but always high temperatures, and their medians demonstrate a weak dependence on temperature. On the contrary, representatives of Methanomicrobia live under similar temperatures but have a high variation of all medians; species of the *Methanosarcina* genus substantially contribute to this variation of medians with a quite similar environmental temperature. Species living under significantly different temperatures and with significantly different medians occur in the classes Methanobacteria and Methanococci.

Convergent genes often overlap in many hyperthermophiles. At low temperatures, it can cause competition between RNA polymerases in the course of elongation. This effect is confirmed by modeling in plastids [18]. The elongation rate of RNA polymerases increases with temperature [20, 21], which reduces the probability of interference of RNA polymerases. Accordingly, the overlapping of convergent genes has a smaller impact on the rate of their transcription.

### 3.3. Bacteria

In bacteria, the overlapping of unidirectional or convergent neighboring genes is typical for mesophiles and thermophiles but not for psychrophiles. The greatest medians of any type correspond to mesophiles (Figure 1). This effect can be due to a small number of considered bacterial species living at low temperatures. However, high median levels are not observed in considered thermophiles, as in archaea.

## 4. Conclusions

A large volume of data from plastids of the rhodophytic branch as well as complete archaeal and bacterial genomes was used to demonstrate the following uniform and statistically significant patterns. The median distances between convergent, divergent, and unidirectional neighboring genes are linearly related to each other. The optimal growth temperature and each of the three medians are exponentially related. The equations relating the medians as well as the optimal growth temperature to each median have their leading coefficients of the same sign and order of magnitude, which can indicate a universal pattern of these relationships. Similar results for such distant genomes are prominent at the qualitative level as well. For instance, the medians are low at high temperatures. At low temperatures, the medians tend to statistically significant greater values and scattering. We propose that changes in environmental temperature optimize intergenic distances, among other things, to decrease the competition of RNA polymerases within the locus that results in transcribing shortened RNAs. Overall, this points to an effect of temperature for both remote and close genomes.

## 5. Methods

Plastid, archaean, and bacterial genomes were extracted from GenBank, and the OGT values are taken from [34] for Archaea, [34–38] for Bacteria, and the publications [7, 28–30, 39–87] for plastids (see Supplementary Information). They are listed in the Supplementary Spreadsheet along with OGT data obtained from the experiment-based papers. *Growth* temperatures are considered as temperatures permissible for the growth of particular cell culture, while *optimal* temperatures provide for the optimal cell culture growth.

## Data Availability

All data generated or analyzed during this study are included in this article and Supplementary Materials.

## Supplementary Materials

Supplementary Materials Supplementary Spreadsheet.xlsx: this Microsoft Office Spreadsheet (cited as Supplementary Spreadsheet) contains the source data: median intergenic distances and optimal growth temperatures. Each row contains the following: sequence accessions; medians *con*, *div*, and *uni*; optimal growth temperature; OGT; and species name. The medians are calculated for the specified set of genomic sequences in total. The OGT calculation is explicated in the Supplementary Information. Column G contains the suggested partition of the data by OGT. Supplementary Materials.pdf: this document (cited as Supplementary Materials) provides more details on the methods and some auxiliary results. Supplementary Information.pdf: this document (cited as Supplementary Information) contains additional information on optimal growth temperature and other habitation conditions of species.
