# Supplementary material for: Optimal Growth Temperature and Intergenic Distances in Bacteria, Archaea, and Plastids of Rhodophytic Branch
Source: Biomed Res Int. 2020 Jan 17;2020:3465380. doi: 10.1155/2020/3465380 (PMC6991167; doi:10.1155/2020/3465380)
Supplement: Supplementary Materials — Supplementary Spreadsheet.xlsx: this Microsoft Office Spreadsheet (cited as Supplementary Spreadsheet) contains the source data: median intergenic distances and optimal growth temperatures. Each row contains the following: sequence accessions; medians con, div, and uni; optimal growth temperature; OGT; and species name. The medians are calculated for the specified set of genomic sequences in total. The OGT calculation is explicated in the Supplementary Information. Column G contains the suggested partition of the data by OGT. Supplementary Materials.pdf: this document (cited as Supplementary Materials) provides more details on the methods and some auxiliary results. Supplementary Information.pdf: this document (cited as Supplementary Information) contains additional information on optimal growth temperature and other habitation conditions of species. [file 3465380.f1.zip › Supplementary Information.pdf]

Supplementary Information for the Article
“Optimal Growth Temperature and Intergenic Distances in Bacteria, Archaea, and Plastids of Rhodophytic Branch”

by Vassily A. Lyubetsky, Oleg A. Zverkov, Lev I. Rubanov, and Alexandr V. Seliverstov

Information on optimal growth temperature and other habitation conditions of species

The optimal growth temperature (OGT) varies in a wide range for different species.

The microalgae *Nannochloropsis limnetica* has adapted to a freshwater environment with 0.6°C [51]; however, its OGT is 15°–22°C [54]. The same OGT is observed in marine alga *Nannochloropsis gaditana* [54]. Other tested *Nannochloropsis* species, *N. oceanica* CCMP531, *N. salina* CCMP537, *N. gaditana* CCMP527, *N. oculata* CCMP525, *N. limnetica* CCMP505, *N. granulata* CCMP529, and *N. oceanica* have similar plastid genomes and grow at 25°C [85].

The alga *Trachydiscus minutus* grows at 20–32°C, while its growth decelerates and the chlorophyll content decreases at 15°C or 40°C [56].

Many diatoms (Bacillariophyceae) can photosynthesize at low temperature. As a result, they can dominate in cold water in spring in the regions with seasonal temperature variations. However, the diatom *Fistulifera solaris* (JPCC DA0580) grows at 20–35°C [81]. The OGT for *Thalassiosira pseudonana* and *Pseudo-nitzschia fraudulenta* is about 25 and 21°C, respectively [48].

The unicellular alga *Triparma laevis*, a relative of diatoms, grows at 0–10°C and does not occur at temperatures of 15°C and above [58, 69].

The OGT for the yellow-green alga *Vaucheria litorea* is 15°–20°C [74, 39].

For many brown algae (Phaeophyceae), the OGT is 10°–15°C. In particular, it equals 10°C for *Laminaria longicruris* and *L. digitate* and 15°C for *L. hyperborea*; *L. saccharina* demonstrates optimal growth at 10°–15°C; and the maximum growth temperature for *Laminaria* species ranges from 18 to 23°C [41]. The OGT for *Saccharina japonica* is about 15°C, and its growth substantially decelerates as temperature increases to 25°C [69]. The OGT for the brown algae *Ectocarpus siliculosus* strain Ec32 (CCAP1310/4) used for the sequencing of plastid DNA [67] is 15°C [78]. The growth rate of certain *Ectocarpus siliculosus* isolates has an acute maximum at 15°C, although two isolates demonstrate a diffuse peak at 15–20°C; and these algae die at temperatures above 26°C [40]. Another brown alga, *Fucus vesiculosus*, grows at 10°–24°C; and dies at 27°C [57]. In this case, the OGT substantially varies for algae from different locations. The OGT for the Baltic *F. vesiculosus* ranges from 4 to 10°C,

while that for *F. vesiculosus* from the Irish Sea is 15–20°C [76]. The average increase in the growth rate for *F. vesiculosus* at 10, 12.5, and 15°C was found to be 280% that at 7°C [83].

Certain brown algae die at temperatures optimal for cyanobacteria. At the same time, brown algae of the *Sargassum* genus live at higher temperatures. The OGT is 15°C for *Sargassum muticum* [87], 15°–20°C for *S. thunbergii* [62], 20°C for *S. patens* [50], and 25°C for *S. horneri* [46] and *S. henslowianum* [45]. The OGT for another brown alga *Undaria pinnatifida* living in the Okirai Bay and the Matsushima Bay is 14°–16°C, and it dies at 22°C. The OGT for this species from the Naruto Strait is 18°C; however, they can survive higher temperatures [55]. Wehr *et al.* [84] described *Pleurocladia lacustris* populations growing at 9–12°C. *P. lacustris* was reported near many Arctic islands [86]. Imbs *et al.* [60] studied seasonal changes in the chemical composition of the brown alga *Costaria costata*. Nakabayashi and Taniguchi [75] studied seasonal changes in another brown alga, *Coccophora langsdorfii*. Its eggs were released from receptacles when the sea surface temperature exceeded 8°C in April. After maturation, the thalli gradually withered and shed in spring and summer, and decayed away in November, two years after the appearance.

The algae *Chromera velia* CCMP2878 and *Vitrella brassicaformis* CCMP3155 (Alveolata) live in warm water up to 28°C with the OGT being about 22°C [6]. Alveolates also include photosynthetic dinoflagellates. *Kryptoperidinium foliaceum* typically divide at 19°–23°C and less frequently at 15°C [53]. *Durinskia baltica* also lives at relatively high temperatures from 20 to 25°C [68]. The OGT for *Karlodinium veneficum* (Norwegian isolate) ranges from 20 to 24°C, although it can grow at temperatures from 7 to 24°C [79]. The OGT for *Lepidodinium chlorophorum* is about 22°C [48]. The non-photosynthetic cryptomonad *Cryptomonas paramecium* strain 977/2a can be maintained at room temperature [49].

The rate of *Emiliania huxleyi* growth increases with temperature from 2 to 27°C, but its growth ceases at temperatures above 27°C [52]; the OGT for *E. huxleyi* is in the range from 22 to 25°C [48]. Another haptophyte, *Pavlova lutheri* grows at temperatures from 10 to 26°C [44] with the OGT being 22°–23°C [48]. *Phaeocystis antarctica* proliferates in the Ross Sea (Antarctica) at temperatures starting from -1.8°C [82]. A wide range of growth temperatures was reported for *Phaeocystis globosa*, from 4 to 30°C [70].

The OGT for *Heterosigma akashiwo* is 25°C; however, they can live in much colder water and the alga becomes more dangerous for fish at low temperatures [77].

The OGT for *Aureococcus anophagefferens* ranges from 20 to 25°C [42]. The alga *Aureoumbra lagunensis* grows much faster at 25°C than at 15°C [43].

The OGT for cyanobacteria is usually above 25°C [47, 39]. For instance, *Microcystis* grow very slowly at temperatures below 13°–15°C and their OGT is 27°C [80].

The algae *Bangia* spp. and *Porphyra* spp. have a complex life cycle with stages alternating depending on temperature and light period. The life cycle of many species is linked to seasonal periodicity. The OGT for *P. lacerata* Miura [73], *P. moriensis* Ohmi [72], *P. pseudolinearis* Ueda, and *P. dentata* Kjellman [63] is 5–15°C for the blades and 20°C for the conchocelis phase. The optimal temperature for conchosporangia formation is 25°C in *Porphyra yezoensis* HB (*Pyropia yezoensis*) [28]. In *P. columbina*, conchosporangia are formed at 10–15°C depending on the light period, while the growth of juvenile fronds peaked at 15°C [29]. The freshwater alga *Batrachospermum turfosum* (Florideophyceae) is capable of net photosynthesis at temperatures between 5°C and 35°C [30].

Parasites of homeotherms live at about 37°C. Among them, *Leucocytozoon caulleryi, Plasmodium chabaudi,* and *P. vivax* are insect-borne: *Culicoides arakawae* transmits *L. caulleryi* and mosquitoes transmit *Plasmodium* spp. On the contrary, coccidians *Cyclospora cayetanensis, Eimeria tenella,* and *Toxoplasma gondii* occur only in warm-blooded animals.

Algae of the class Cyanidiophyceae (Rhodophyta) live at high temperature. We considered three genera of the class: *Cyanidium, Galdieria,* and *Cyanidioschyzon*. *Cyanidioschyzon merolae* lives at about 45°C [71] and can survive heat shock at 63°C [66]. The alga *Cyanidium caldarium* can live at diverse temperatures from below 20°C up to 56°C [64, 65]. The alga *Galdieria sulphuraria* proliferates at 50–55°C [61].

Unfortunately, no published data on the OGT could be found for certain species; in such cases supplementary data on the temperature were obtained from the database at https://ncma.bigelow.org/products/algae/all-algae/. These species include *Guillardia theta, Rhodomonas salina, Chrysochromulina parva, Cylindrotheca closterium, Phaeodactylum tricornutum, Chaetoceros simplex, Coscinodiscus radiatus, Thalassiosira oceanica, T. weissflogii, Asterionellopsis glacialis, Odontella sinensis, Lithodesmium undulatum, Ochromonas sp., Toxarium undulatum*.
