# Supplementary material for: Optimal Growth Temperature and Intergenic Distances in Bacteria, Archaea, and Plastids of Rhodophytic Branch
Source: Biomed Res Int. 2020 Jan 17;2020:3465380. doi: 10.1155/2020/3465380 (PMC6991167; doi:10.1155/2020/3465380)
Supplement: Supplementary Materials — Supplementary Spreadsheet.xlsx: this Microsoft Office Spreadsheet (cited as Supplementary Spreadsheet) contains the source data: median intergenic distances and optimal growth temperatures. Each row contains the following: sequence accessions; medians con, div, and uni; optimal growth temperature; OGT; and species name. The medians are calculated for the specified set of genomic sequences in total. The OGT calculation is explicated in the Supplementary Information. Column G contains the suggested partition of the data by OGT. Supplementary Materials.pdf: this document (cited as Supplementary Materials) provides more details on the methods and some auxiliary results. Supplementary Information.pdf: this document (cited as Supplementary Information) contains additional information on optimal growth temperature and other habitation conditions of species. [file 3465380.f1.zip › Supplementary Materials.pdf]

Supplementary Materials for the Article
"Optimal Growth Temperature and Intergenic Distances in Bacteria, Archaea, and Plastids of Rhodophytic Branch"
by Vassily A. Lyubetsky, Oleg A. Zverkov, Lev I. Rubanov, and Alexandr V. Seliverstov

§1. Correlation coefficients for medians and temperature

The *Pearson correlation* of x and y equals $\frac{\sum_i (x_i-\bar{x})(y_i-\bar{y})}{\sqrt{\sum_i (x_i-\bar{x})^2 (y_i-\bar{y})^2}}$, where $\bar{x}$ and $\bar{y}$ are means.

In the six rightmost columns $e(\cdot)$ and $h(\cdot)$ denote the exponential and hyperbolic regression values, respectively, which are discussed in the paper. Highlighted are sounded values.

Table S1.

	c	*d*	*u*	c^2	d^2	u^2	*cd*	*cu*	*du*	*e*(*c*)	*e*(*d*)	*e*(*u*)	*h*(*c*)	*h*(*d*)	*h*(*u*)
							**Plastids**								
OGT	-0.18	-0.46	-0.36	-0.04	-0.18	-0.11	-0.07	-0.06	-0.14	0.65	0.71	0.70	0.60	0.70	0.68
c		0.82	0.88												
d			0.91												
							**Archaea**								
T	-0.62	-0.60	-0.59	-0.48	-0.52	-0.51	-0.51	-0.49	-0.51	0.76	0.75	0.65	0.76	0.73	0.65
c		0.95	0.90												
d			0.93												
							**Bacteria**								
T	-0.18	-0.18	-0.33	-0.07	-0.15	-0.23	-0.14	-0.17	-0.25	0.44	0.24	0.48	0.41	0.23	0.48
c		0.56	0.54												
d			0.64												

§2. To estimate the regression quality and to compare the regression with smaller number of parameters and the regression with the extended parameter set we use the *Fisher index* (Fisher statistic) $\bar{F}_{calc} = \left(\frac{\sum_i (y_i - \hat{z}_i)^2}{\sum_i (y_i - \hat{y}_i)^2} - 1 \right) \cdot \frac{k_2}{k_1}$, where $\hat{z}$ is the regression in the smaller space of functions and $\hat{y}$ is the regression in the larger space of functions, $\hat{z}_i = \hat{z}(x_i)$, $\hat{y}_i = \hat{y}(x_i)$, but k_1 is the difference of dimensions of larger and smaller spaces and k_2 is the difference of data dimension *n* and the dimension of the smaller space of functions. The *Fisher test* demands to verify the inequality $\bar{F}_{calc} > F_{tabl}(\alpha, k_1, k_2)$, where righthand side is the α-quantile of the Fisher distribution. The *radius* of the confidence interval for linear correlation is given below, and mean square errors of the coefficients are half or less of the radii.

Table S2. Regressions between medians of intergenic distances for three types of neighboring gene arrangement. The groups of plastids, archaea, and bacteria are referred to as *P*, *A*, and *B* in column *G*, respectively. The linear simple (left) and Deming (right) regressions are given, for the regression coefficients, 95% confidence interval radii are specified with ±. The standard deviation $\sqrt{\frac{1}{n-k}\cdot\sum_{i=1}^{n}(y_i-\hat{y}(x_i))^2}$ for obtained regressions is designated as s^*, where n is the data dimension. The Fisher index $\bar{F}_{calc}$ is given in comparison with the constants. The tabular values are similar for all three groups: $F_{0.05}(1, 57) \approx F_{0.05}(1, 121) \approx F_{0.05}(1, 808) = 4$. Three rightmost columns specify the proportion of residuals with absolute values not exceeding s^*, $2s^*$, and $3s^*$.

G	*Regression*	s^*	$\bar{F}_{calc}$	$\pm s^*$	$\pm 2s^*$	$\pm 3s^*$
P	$u = (0.19\pm0.03)c + (32.5\pm5)$; $u = 0.20c + 32.3$	16; 16	188; 188	70; 70	98; 97	98; 98
P	$u = (0.28\pm0.03)d + (2.5\pm7)$; $u = 0.29d + 1.7$	14; 14	261; 261	76; 76	97; 97	97; 97
A	$u = (0.66\pm0.06)c + (17\pm5)$; $u = 0.71c + 15$	21; 22	498; 483	76; 78	97; 97	98; 98
A	$u = (0.43\pm0.03)d - (30\pm6)$; $u = 0.44d - 32$	17; 17	815; 811	73; 72	93; 94	100; 100
B	$u = (0.27\pm0.03)c + (27\pm3)$; $u = 0.32c + 23$	19; 20	331; 314	73; 74	96; 96	99; 99
B	$u = (0.29\pm0.02)d - (10\pm5)$; $u = 0.33d - 17$	18; 18	553; 537	69; 70	95; 95	99; 100

§3. For any partition (grouping, clustering, pooling) of the species set onto the *parts* (groups, pools) we calculate another *Fisher index* (statistic) $F_{calc} = \frac{\sum_{i=1}^{k} n_i\left(\bar{x}_i-\bar{x}\right)^2}{\sum_{i=1}^{k}\sum_{j=1}^{n_i}\left(x_{ij}-\bar{x}_i\right)^2}\cdot\frac{n-k}{k-1}$, where k is a number of pools, n_i is a number of members in the i-th pool, $\bar{x}_i$ – the sample mean for the i-th pool, x_{ij} are all sample members, $\bar{x}$ the total sample mean, and n is the data dimension. The Fisher test for the significant difference between the pool mean values is $F_{calc} > F_{tabl}(\alpha, k-1, n-k)$.

For any pair of pools we can compare their mean values using the Student test and the Behrens-Fisher test, which have statistics $\frac{|\bar{x}_1-\bar{x}_2|}{\sqrt{\frac{\sum_{i=1}^{n_1}(x_i-\bar{x}_1)^2+\sum_{i=1}^{n_2}(x_i-\bar{x}_2)^2}{n_1+n_2-2}\cdot(\frac{1}{n_1}+\frac{1}{n_2})}}$ and $\frac{|\bar{x}_1-\bar{x}_2|}{\sqrt{\sum_{i=1}^{n_1}(x_i-\bar{x}_1)^2/((n_1-1)\cdot n_1)+\sum_{i=1}^{n_2}(x_i-\bar{x}_2)^2/((n_2-1)\cdot n_2)}}$. In the first case we need assumption of equal variances and in the second one we do not. The statistics are compared with the Student's α-quantile and with the α-quantile of the normal distribution. Here 1 referred to the first pool in the pair and 2 to the second one. Instead of the Student test, for the same purpose the Fisher test and $F_{tabl}(\alpha, 1, n-2)$ can be used.

To verify an equality of variances we use yet another *Fisher test*

$$\frac{\sum_{i=1}^{n_1}(x_i-\bar{x}_1)^2/(n_1-1)}{\sum_{i=1}^{n_2}(x_i-\bar{x}_2)^2/(n_2-1)} > \max(F_{tabl}(\frac{\alpha}{2},k_1,k_2),F_{tabl}(\frac{\alpha}{2},k_2,k_1))$$

, where the larger sample variance is in the numerator. Here F_{tabl} is the quantile of the Fisher distribution with $k_1 = n_1 - 1$ and $k_2 = n_2 - 2$ degrees of freedom.

Table S3. Comparison of the parts of the partition. For each pair of the parts shown in the Spreadsheet the three column groups (each for medians *con*, *div*, *uni*) show, left to right: the Fisher index with its critical value, the Behrens–Fisher index with 1.96 as the critical value, and the variance ratio with its critical value. Highlighted are the values exceeding the critical levels.

		**Fisher**				**Behrens–Fisher**			**Variance Ratio**			
		con	*div*	*uni*	F_{table}	*con*	*div*	*uni*	*con*	*div*	*uni*	F_{table}
**Plastids**	*G1, G2*	0.09	1.12	5.05	4.28	0.30	1.06	2.20	1.46	1.02	2.73	2.72
	G1, G3	0.39	0.49	3.59	4.15	0.75	0.86	2.02	3.62	5.02	1.55	2.64
	G1, G4	20.81	118.32	64.7	4.32	4.73	10.85	7.93	9.50	1.13	1.91	2.94
	G2, G3	0.74	0.01	0.02	4.14	1.04	0.10	0.18	5.29	5.12	4.22	2.53
	G2, G4	25.07	102.97	60.17	4.30	5.34	10.08	7.31	6.52	1.16	5.22	2.91
	G3, G4	9.70	34.43	45.92	4.16	4.35	7.31	6.53	34.45	4.43	1.24	2.76
**Archaea**	*G1, G2*	7.08	7.14	3.25	4.00	3.66	3.30	2.27	3.89	2.30	2.51	2.25
	G1, G3	86.63	78.47	77.64	3.93	8.25	7.87	7.87	21.97	18.35	12.99	1.57
	G2, G3	36.26	17.92	41.17	3.97	3.80	2.51	4.12	5.64	7.97	5.17	1.86
**Bacteria**	*G1, G2*	2.11	0.29	21.89	3.85	1.5	0.54	4.78	1.65	1.10	1.34	1.19
	G1, G3	0.05	0.07	2.60	3.87	0.22	0.27	1.29	1.05	1.04	1.83	1.42
	G1, G4	54.79	35.91	109.04	3.87	8.05	5.44	10.29	1.27	1.32	1.04	1.45
	G2, G3	0.22	0.00	0.40	3.86	0.56	0	0.56	1.57	1.14	1.37	1.50
	G2, G4	44.62	31.61	48.99	3.86	8.82	5.24	7.7	2.09	1.20	1.29	1.45
	G3, G4	33.37	15.81	28.53	3.94	5.7	4.03	5.21	1.34	1.37	1.76	1.60

The Fisher index makes it possible to evaluate the pairwise similarity and difference between the groups, which will be referred to as *G1, G2, G3,* and *G4* in ascending temperature order, respectively. In plastids, the *G4* group markedly differs from *G1* and *G2*, and partially from G3; while the difference between *G1, G2,* and *G3* is less pronounced or even insignificant. In archaea, *G3* sharply differs from *G1* and *G2*, while the difference between *G1* and *G2* is not as pronounced. In bacteria, G4 sharply differs from other groups, the difference between which is lower or even

insignificant (for numerical evaluations, see Table S3). The Behrens–Fisher test was used since the variance equality for the groups is not always significant, and this test does not require it. The second test yields similar results except for archaea, for which all groups significantly differ. According to the variance analysis, *G4* is sharply distinct from *G3* by *con* in plastids; *G3* is sharply distinct from *G1* and less distinct from other groups in archaea; while the differences between groups are minor or even insignificant in bacteria (Table S3). Thus, the clustering into four groups for plastids and bacteria meets only certain pairwise comparison tests. A special situation is observed for the first test: it tends to combine *G1* and *G3* or *G2* and *G3*, while *G1* and *G2* notably differ. A similar pattern is observed for the second test in the case of bacteria, while *G2* and *G3* can be combined for plastids, which yields three groups. The clustering into two parts gives the maximum Fisher index for the temperatures of about 30°C and 40-60°C in the case of plastids and archaea or for 60-65°C in bacteria (Table S3). This generally corresponds to the division into mesophiles and thermophiles.

§4. To compare linear and non-linear regressions we used *Bayesian information criterion*: $BIC = n\cdot\ln(\sum_{i=1}^{n}(T_i-\widehat{T}(x_i))^2)+(k-n)\cdot\ln n$, where *n* is a number of data points and *k* is a number of the regression parameters. The Bayes factor Δ*BIC=BIC1 – BIC2* defines the strength of the evidence against the linear regression with the non-linear one. It can be summarized as follows: from 6 to 10 means "strong", more than 10 means "very strong". The quality of the regression curve (explained variation, *determination coefficient* R^2) is calculated as $1-\frac{\sum_{i=1}^{n}(y_i-\widehat{y}_i)^2}{\sum_{i=1}^{n}(y_i-\overline{y})^2}$ in percentage. The coefficients of exponential and hyperbolic regressions were found by the numerical method implemented programmatically. Specifically, we sought for absolute minimum of the standard deviation error function *s* for a set of experimental observations (points). For that purpose, we established a grid of initial points in one- or three-dimensional real space. From each initial point, a steepest descent was applied as follows: the gradient *s* is estimated at the current point then the first local minimum is sought in the direction of the negative gradient, which becomes the next point, and so on until the module of the gradient is less than $\varepsilon=10^{-9}$. The ultimate result of such minimization is a final point with the least value of *s* among those reached from all initial points. Together with seeking for the optimum (best-fitting) regression, the program stores a regression with best s and residuals satisfying the *Pearson goodness-of-fit test* $\chi^2=\sum_{i=1}^{l}\frac{(n_i-np_i)^2}{np_i}<\chi^2_{tabl}(\alpha,l-3)$, where *l* is an intervals number, n_i is a number of sample members in *i*-th interval, n is the sample cardinality, p_i is the theoretical probability to belong to the *i*-th interval according to the normal distribution with the mean and the variance estimated from the sample. The threshold in the righthand side is the α-quantile of the χ^2-distribution.

Table S4. Polynomial regressions for OGT and medians in plastids, archaea and bacteria. The $\bar{F}_{calc}$ column shows the Fisher index comparing each polynomial of degree *n* to the polynomial of degree (*n* – 1). The next two columns show the Bayesian information criterion value *ΔBIC* and the percent of explained variance R^2. For any linear regression coefficient, 95% confidence interval radii are specified with ±. The rightmost columns characterize the residuals distributions. For plastids and archaea they pass Pearson's chi-squared test. For bacteria, they do not pass Pearson's test; therefore the last three columns show the percentage of residuals within ±*s**, ±2*s** и ±3*s** intervals where *s** is their standard deviation shown in the *s* column. The table values are shown next to the group name. Ref. also to Table S5.

Plastids: $F_{0.05}(1, 55–57) = 4$; $F_{0.05}(3, 55) = 2.8$, $F_{0.05}(6, 49) = 2.3$, $F_{0.05}(10, 39) = 2.1$.

Regression	*s* (°C)	$\bar{F}_{calc}$	*ΔBIC*	R^2	$\mathcal{N}$
$T = (-0.011 \pm 0.016)c + (23.9 \pm 2.9)$	9.4	2	269	3	+/–
$T = 0.000064c^2 - 0.072c + 28$	8.7	10	264	18	+
$T = (-0.04 \pm 0.02)d + (29.8 \pm 4.2)$	8.5	16	257	21	+
$T = 0.00016d^2 - 0.134d + 40$	6.8	32	234	50	–
$T = (-0.1 \pm 0.07)u + (28 \pm 4.3)$	8.9	9	263	13	+/–
$T = 0.0016u^2 - 0.5u + 43$	6.9	41	237	48	+/–

Archaea: $F_{0.05}(1, 119–121) = 3.9$; $F_{0.05}(3, 119) = 2.7$, $F_{0.05}(6, 113) = 2.2$, $F_{0.05}(10, 103) = 1.9$.

Regression	*s* (°C)	$\bar{F}_{calc}$	*ΔBIC*	R^2	$\mathcal{N}$
$T = (-0.23 \pm 0.05)\ c + (74 \pm 4.3)$	18.8	77	729	39	–
$T = 0.0021c^2 - 0.67c + 81$	18.5	38	699	54	–
$T = (-0.14 \pm 0.03)d + (88 \pm 7.1)$	19.3	68	734	36	–
$T = 0.001d^2 - 0.69d + 145$	16.5	45	700	53	–
$T = (-0.295 \pm 0.07)u + (77 \pm 5.1)$	19.4	65	736	35	+/–
$T = 0.0028u^2 - 0.76u + 87$	18.5	13	728	41	–

Bacteria: $F_{0.05}(1, 806–808) = 3.9$; $F_{0.05}(3, 806) = 2.6$, $F_{0.05}(6, 800) = 2.1$, $F_{0.05}(10, 790) = 1.8$.

Regression	*s* (°C)	$\bar{F}_{calc}$	*ΔBIC*	R^2	±*s*	±2*s*	±3*s*
$T = (-0.043 \pm 0.017)c + (38.5 \pm 1.5)$	11.1	26	3903	3	82.1	91.5	97.5
$T = 0.00039c^2 - 0.134c + 42.4$	10.8	37	3877	7	81.4	91.2	97.9
$T = (-0.041 \pm 0.016)d + (43.3 \pm 3.1)$	11.0	28	3901	3	83.0	91.6	97.4
$T = 0.00041d^2 - 0.204d + 58.4$	10.9	18	3890	5	82.5	91.0	97.0
$T = (-0.16 \pm 0.03)u + (43 \pm 1.7)$	10.6	97	3836	11	82.2	91.4	98.3
$T = 0.0036u^2 - 0.542u + 51$	10.2	67	3779	18	80.1	90.9	98.4

Table S5. Exponential regressions between each of the medians of three types of intergenic distances and OGT. Temperature OGT is designated as *T*. The standard deviation for obtained regressions is designated as s^*. If the presented regression is suboptimal, the s^* value is given in column *s* in parentheses. The Fisher index $\bar{F}_{calc}$, the Bayes factor *ΔBIC=BIC1 – BIC2*, and the proportion R^2 of the explained variance are given in the corresponding columns (in percent). The percentage of residuals whose absolute value does not exceed *s*, 2*s*, and 3*s* are given in last three columns.

Regression	*s*	$\bar{F}_{calc}$	*ΔBIC*	R^2	±*s*	±2*s*	±3*s*
**Plastids**							
$T = 20.3\exp\{-0.022c\} + 16.6$	7.34	20	26	42	76	95	98
$T = 1193/(c+52.5)+12$	7.71	16	20	36	78	95	98
$T = 3.18\cdot10^6(c+195)^{-2} - 16300(c+195)^{-1} + 37$	7.31	14(7)	23	43	78	95	98
$T = 38\exp\{-0.018d\} + 17.3$	7.00 (6.72)	25	19	47	86	95	98
$T = 1200/(d+19.8)+13.7$	6.93 (6.81)	26	20	48	86	93	98
$T = 17.4\cdot10^6(d+355)^{-2} - 49100(d+355)^{-1} + 50.3$	6.93 (6.70)	18(3)	17	49	85	95	98
$T = 21\exp\{-0.035u\} + 18.1$	7.43 (6.90)	19	18	40	81	93	98
$T = 551/(u+17.8)+13.6$	7.44 (7.04)	19	18	40	81	93	98
$T = 6.7\cdot10^6(u+162)^{-2} - 47700(u+162)^{-1} + 96.3$	7.23 (6.86)	15(4)	18	45	83	93	98
**Archaea**							
$T = 50.9\exp\{-0.0256c\} + 34.8$	15.65 (15.62)	83	42	58	74	93	98
$T = 2290/(c+38.1)+24.8$	15.75	81	40	58	71	95	99
$T = 1.15\cdot10^6(c+104)^{-2} - 6700(c+104)^{-1} + 44.2$	15.74 (15.71)	55(1.6)	36	58	73	94	98
$T = 262\exp\{-0.0155d\} + 34.8$	15.93	78	43	57	67	95	99
$T = 5196/(d-21)+21.5$	16.47 (16.43)	69	35	54	65	96	99
$T = 64.2\cdot10^6(d+288)^{-2} - 209500(d+288)^{-1} + 205$	15.92	53(9)	39	57	71	95	98
$T = 58.2\exp\{-0.0178u\} + 31.7$	18.43 (18.38)	43	9	42	72	95	99
$T = 2454/(u+26.9)+20.2$	18.63 (18.38)	41	6	41	76	96	99
$T = 3.8\cdot10^6(u+164.91)^{-2} - 16300(u+164.91)^{-1} + 49.3$	18.50 (18.45)	29(0.1)	4	42	72	95	99

Regression	*s*	$\bar{F}_{calc}$	*ΔBIC*	R^2	±*s*	±2*s*	±3*s*
		**Bacteria**					
$T = 22\exp\{-0.075c\} + 34$	10.08	98	144	20	81.7	91.4	97.7
$T = 384/(c+17.6)+30$	10.23	83	120	17	81.0	91.6	98.1
$T = 115000(c+45)^{-2} - 1900(c+45)^{-1} + 41$	10.09	65(23)	136	19	81.7	91.5	97.7
$T = 41\exp\{-0.015d\} + 32$	10.91	25	14	6	82.6	91.0	96.8
$T = 2640/(d+60)+24.4$	10.95	22	8	5	82.3	91.4	96.9
$T = 43.4\cdot 10^6(d+410)^{-2} - 131000(d+410)^{-1} + 132$	10.91	17(7)	8	6	82.2	91.5	97.0
$T = 37\exp\{-0.089u\} + 33$	9.88	118	110	23	81.4	91.9	98.0
$T = 281/(u+5.7)+28$	9.88	119	111	23	81.5	91.7	97.7
$T = 12500(u+16)^{-2} - 80(u+16)^{-1} + 32$	9.87	80(2)	106	23	81.6	91.7	98.1

We considered both *optimal* regressions, where the minimized function *s* reaches the minimum s^*, and *suboptimal* regressions, where the difference between *s* and s^* does not exceed 10%. This is because an optimal regression can be not with normality property; in this case a normal suboptimal regression was taken. The actual differences between *s* and s^* are smaller in suboptimal regressions: <0.5% in bacteria and <1.5% in archaea; in plastids, the highest differences of 7.7%, 5.6%, and 5.3% are observed for *T*(*u*), otherwise the difference is <5% and <1.5% on average. The obtained regression values are presented in the above table and Fig. 2 in the main text. According to the standard deviation *s*, which was minimized, exponential dependence was always superior to hyperbolic one for both optimal and suboptimal regressions. Suboptimal regressions for plastids and archaea feature the normality property, which we consider as advantageous over optimal regressions. No normality is observed in bacteria for both optimal and suboptimal regressions. Specifically, suboptimal normal regressions was searched as deviated from *s** by <2% in plastids, <1% in archaea, and <10% in bacteria.

For regressions between *T* and *each median*, exponential dependence was superior to hyperbolic one with the only exception of *T*(*uni*) in bacteria, where the *s** values are near equal. The double hyperbolic dependence was almost always superior to the hyperbolic one (with a single exception of *T*(*uni*) in archaea) but, despite a higher number of parameters, is inferior to the exponential dependence for *T*(*con*) and *T*(*uni*) in archaea and *T*(*con*) in bacteria. The regressions in plastids have the following relation (denoted by > according to higher value of *s**): hyp > exp > hyp2. For each class of functions, i.e., separately for hyperbolic, exponential, and double hyperbolic ones, we observe: *con* > *uni* > *div*. In archaea, the relation hyp > exp > hyp2 is valid only for the regressions for *div,* while for the regressions for *con* and *uni* we have hyp > hyp2 > exp. Also we observe *uni* > *div* > *con* for each function class. In bacteria, we have hyp > hyp2 > exp for the regressions for *con* and *div* or exp > hyp > hyp2 for *T*(*uni*). As well as *div* > *con* > *uni* for each function class.

Thus, each group features a unique order of the medians by descending optimal approximation error *s*. Notice that all three possible relations have been realized so that each median occurs once at each position.
